# Use of Anti-Interleukin-6 Receptor Monoclonal Antibody in Drug-Induced Acute Respiratory Distress Syndrome

**DOI:** 10.1155/2020/8832986

**Published:** 2020-07-22

**Authors:** Alessandra Petrillo, Noa Biran, Sean Sadikot

**Affiliations:** Hackensack University Medical Center, Hackensack, NJ, USA

## Abstract

Acute respiratory distress syndrome (ARDS) is a disorder that involves the activation of alveolar macrophages triggering the innate immune system. The parenchymal lung injury seen in ARDS is a result of many proinflammatory elevations including interleukin-6. There remains no effective standard of care of ARDS, and current treatments at this time currently do not target the immunological mechanisms or pathways involved. Treatments involving this pathway should be further investigated as targeted treatment. We discuss a case of a patient with multiple myeloma who was hospitalized with drug-induced ARDS who had a rapid response to an anti-interleukin-6 monoclonal antibody.

## 1. Introduction

Acute respiratory distress syndrome (ARDS) is a disorder for which there is no standard of care therapy and with a mortality rate of 27%, 35%, and 45% for mild, moderate, severe disease, respectively [1]. It is mediated via a systemic inflammatory response involving all organ systems. The innate immune system elicits a systemic response in ARDS involving neutrophils, macrophages, and dendritic cells [2]. Alveolar macrophages lead to the elevation of inflammatory cytokines including interleukin-1beta (IL-1B), tumor necrosis factor-alpha (TNF-a), interleukin-6 (IL-6), and interleukin-8 (IL-8) through the recruitment of neutrophils and macrophages in response to injury [2]. This inflammatory response leads to damaged lung epithelia and endothelia resulting in impaired alveolar-capillary barrier. Accumulation of protein-rich fluid accumulates in alveoli causing impaired gas exchange, subsequently leading to hypoxemia [2]. Current anti-inflammatory therapies that have been investigated for the treatment of ARDS include most commonly corticosteroids [3]. Other treatments have also been used, which include neutrophil elastase inhibitors [4], granulocyte-macrophage colony-stimulating factor (GM-CSF) [5], statins [6], omega-3 fatty acids [7], surfactant [8], inhaled B-agonists [9], and nitric oxide [10]. All treatments mentioned have not shown a mortality benefit. Supportive therapies such as mechanical ventilation [11] and prone positioning [12] are currently the only management for ARDS at this time [2].

Tocilizumab is a humanized monoclonal antibody to IL-6 receptor currently FDA approved for patients with rheumatoid arthritis, giant cell arteritis, systemic juvenile idiopathic arthritis, polyarticular juvenile idiopathic arthritis, and cytokine release syndrome in the setting of chimeric antigen receptor (CAR) T cells [13, 14]. Here, a patient with drug-induced ARDS and pneumonitis with multiorgan failure secondary to a chemotherapeutic agent carfilzomib experienced a rapid and clinically significant resolution of drug-induced ARDS after tocilizumab therapy.

## 2. Case Report

The patient is a 62-year-old female with a past medical history of hypothyroidism who was diagnosed with free kappa multiple myeloma, Durie-Salmon IIIA Revised International Staging System (R-ISS), in 2019 after she presented with acute kidney injury and multiple vertebral compression fractures. Criteria for Durie-Salmon IIIA include one or more of the following: hemoglobin < 8.5 g/dL, serum calcium value > 12 mg/dL, advanced lytic lesions, or high M-component production rates IgG value > 7 g/dL and IgA value > 5 g/dL. Class A refers to a relatively normal renal function or serum creatinine value < 2.0 mg/dL [15].

The patient received induction therapy with carfilzomib (a second-generation proteasome inhibitor), cyclophosphamide (an alkylating agent), and dexamethasone in a twice weekly dosing schedule in a 28-day cycle. She completed her first full cycle without complication. During cycle 2, the cyclophosphamide was replaced by the oral immunomodulatory agent lenalidomide. On cycle 2 day 10, she was admitted with a fever of 102.5°F in respiratory distress requiring nasal cannula. Computed tomography (CT) chest at that time revealed patchy bilateral ground glass opacities consistent with pneumonia versus pulmonary edema. The respiratory pathogen panel was negative, an infectious etiology was not found, and she responded very quickly to stress dose steroids and was discharged two days later. Treatment was restarted a week later. The evening after rechallenge of carfilzomib, lenalidomide, and dexamethasone (cycle 3 day 1), she was found to be in acute respiratory failure by her husband and upon arrival in the emergency department she was found to have Po2 of 54, BP 93/58, and WBC 31.3.

Chest X-ray at the time of presentation showed new extensive multilobar airspace disease related to pneumonia or edema. Despite a trial of bilevel positive airway pressure and the rapid initiation of high-dose steroids and empiric antibiotics, the patient required intubation for respiratory distress. CT chest was consistent with complete opacification of both lungs. She experienced high fevers and multiorgan failure requiring 3 vasopressors, nitric oxide with prone positioning, and hemodialysis. Physical exam was significant for anisocoria and coarse breath sounds bilaterally. Laboratory evaluation revealed WBC 35,000; ferritin 2,500; CRP 17.7; and negative blood cultures. The patient was subsequently administered tocilizumab 500 mg IV 100 mL/hr over 60 minutes 1x. Within 24-48 hrs, vasopressor requirements lessened, anisocoria resolved, oxygen requirements improved, and laboratory evaluation revealed WBC 24,000; CRP 3.4; and ferritin 1000. Her chest X-ray showed significant improvement in diffuse airspace opacification. She was eventually extubated and is currently doing well.

## 3. Discussion

Current treatments for ARDS involve mechanisms which reduce shunt fraction, increase oxygen delivery, decrease oxygen consumption, and avoid further injury [1]. Mechanical ventilation with low tidal volume and high positive end-expiratory pressure (PEEP) and proning remain the most common and standard of care treatments for severe ARDS. Low tidal volume reduces lung stretch along with a reduction in inflammatory cytokines [11]. High PEEP is used to reduce lung collapse at end expiration and improve oxygenation [1]. However, methods to determine selection of the optimal PEEP level without leading to injury and lung overdistention is still unclear [1]. Prone positioning helps to alleviate lung compression from mediastinal and abdominal structures, improves oxygenation through redistribution of lung edema to less perfused areas, and reduces transpulmonary pressures that lead to tissue injury [1]. However, prone positioning is associated with increased risk of adverse events such as pressure ulcers, endotracheal obstruction, and catheter dislodgement [1]. Of these treatments, the mortality of ARDS remains at 45% for severe cases.

**Figure 1:**
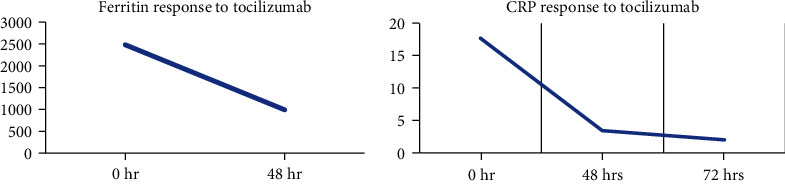
Inflammatory markers ferritin and CRP were both measured prior to and after the administration of an anti-IL-6 receptor monoclonal antibody. Administration of tocilizumab shows a time-dependent decrease in both ferritin and CRP within 48-72 hours. Baseline ferritin decreased from 2,500 to almost 1,000, whereas baseline CRP decreased from 17 to 2.0.

**Figure 2:**
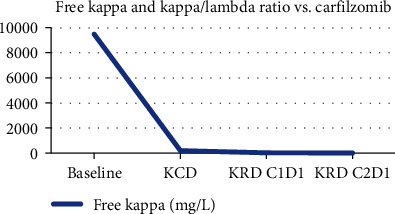
Multiple myeloma marker responses to carfilzomib. Demonstration of cycle-dependent decline in free kappa and kappa/lambda ratios.

**Figure 3:**
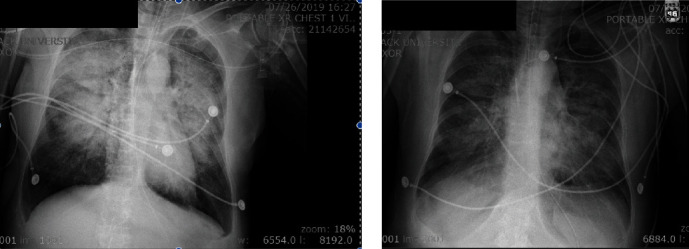
Chest X-ray prior to and following tocilizumab. Improvement of bilateral ground glass opacities following interleukin-6 receptor inhibitor.

The case presented here involves a patient with drug-induced ARDS who demonstrated a response with an IL-6 receptor inhibitor as seen with her clinical improvement and measured with ferritin and CRP (Figure 1) At this time, her multiple myeloma was well controlled with carfilzomib as seen in (Figure 2) with a dose-dependent decrease in myeloma marker free kappa chain. In addition, her chest X-ray showed dramatic improvement after tocilizumab (Figure 3). She was discharged from the ICU within 48 hours of treatment. Her recovery was unanticipated given her initial severe presentation.

## 4. Conclusion

The patient in this case was in multiorgan failure at the time and had poor response to steroids. This left the patient without options for treatment of her drug-induced ARDS, and therefore, an IL-6 receptor inhibitor was considered to reduce the inflammatory response.

Treatment for ARDS should be centered around a targeted approach to the inflammatory mediators involved in the process. IL-6 plays a key role in leading to endothelial injury subsequently causing hypoxemia. Targeting IL-6 levels through means of IL-6 receptor inhibition would therefore assist in mechanisms for treatment to prevent cytokine-induced injury that subsequently leads to ARDS. Tocilizumab has also been used in Still's disease complicated with ARDSs and most recently in SARS-CoV-2 (COVID-19)-induced ARDS [16, 17]. It can be hypothesized, based on the clinical response seen in this patient, that tocilizumab played a critical role in modulating the pathophysiological immune response of ARDS. Therefore, tocilizumab should be investigated in the future for the targeted treatment of inflammatory diseases aside from cytokine release syndrome, such as ARDS.

## Data Availability

No additional data is available.

## Abbreviations

CAR: Chimeric antigen receptor.

## References

[1] Diamond M., Peniston Feliciano H. L., Sanghavi D., Mahapatra Sidharth (2020). Acute respiratory distress syndrome (ARDS) [Updated 2020 Jan 5].. *StatPearls [Internet]*.

[2] Han S., Mallampalli R. K. (2015). The acute respiratory distress syndrome: from mechanism to translation. *Journal of Immunology*.

[3] Peter J. V., John P., Graham P. L., Moran J. L., George I. A., Bersten A. (2008). Corticosteroids in the prevention and treatment of acute respiratory distress syndrome (ARDS) in adults: meta-analysis. *BMJ*.

[4] Iwata K., Doi A., Ohji G. (2010). Effect of neutrophil elastase inhibitor (sivelestat sodium) in the treatment of acute lung injury (ALI) and acute respiratory distress syndrome (ARDS): a systematic review and meta-analysis. *Internal Medicine*.

[5] Paine R., Standiford T. J., Dechert R. E. (2012). A randomized trial of recombinant human granulocyte-macrophage colony stimulating factor for patients with acute lung injury. *Critical Care Medicine*.

[6] The National Heart, Lung, and Blood Institute ARDS Clinical Trials Network (2014). Rosuvastatin for sepsis-associated acute respiratory distress syndrome. *The New England Journal of Medicine*.

[7] Rice T. W., Wheeler A. P., Thompson B. T. (2011). Enteral omega-3 fatty acid, *γ*-linolenic acid, and antioxidant supplementation in acute lung injury. *JAMA*.

[8] Anzueto A., Baughman R. P., Guntupalli K. K. (1996). Aerosolized surfactant in adults with sepsis-induced acute respiratory distress syndrome. *The New England Journal of Medicine*.

[9] The National Heart, Lung, and Blood Institute Acute Respiratory Distress Syndrome (ARDS) Clinical Trials Network (2011). Randomized, placebo-controlled clinical trial of an aerosolized *β*_2_-Agonist for treatment of acute lung injury. *American Journal of Respiratory and Critical Care Medicine*.

[10] Dellinger R. P., Zimmerman J. L., Taylor R. W. (1998). Effects of inhaled nitric oxide in patients with acute respiratory distress syndrome. *Critical Care Medicine*.

[11] The Acute Respiratory Distress Syndrome Network (2000). Ventilation with lower tidal volumes as compared with traditional tidal volumes for acute lung injury and the acute respiratory distress syndrome. *The New England Journal of Medicine*.

[12] Guérin C., Reignier J., Richard J.-C. (2013). Prone positioning in severe acute respiratory distress syndrome. *The New England Journal of Medicine*.

[13] National Cancer Institute NCI Drug Dictionary. http://www.cancer.gov/publications/dictionaries/cancer-drug/def/tocilizumab.

[14] GCA, SJIA, PJIA Treatment | ACTEMRA® (Tocilizumab) Learn more about Actemra. https://www.actemra.com.

[15] International Myeloma Foundation Durie-Salmon staging system. http://www.myeloma.org/durie-salmon-staging.

[16] Kaneko Y., Kameda H., Ikeda K. (2018). Tocilizumab in patients with adult-onset Still's disease refractory to glucocorticoid treatment: a randomised, double-blind, placebo-controlled phase III trial. *Annals of the Rheumatic Diseases*.

[17] Campochiaro C., Della-Torre E., Cavalli G. (2020). Efficacy and safety of tocilizumab in severe COVID-19 patients: a single- centre retrospective cohort study. *European Journal of Internal Medicine*.

